# A Rare Case of Mycobacterium Monacense Osteomyelitis: A Case Report

**DOI:** 10.7759/cureus.14199

**Published:** 2021-03-31

**Authors:** Shoaib Ashraf, Christian Mendoza, Syeda Hafsah Salman, Paul Kelly, Muhammad Adrish

**Affiliations:** 1 Internal Medicine, BronxCare Health System/Icahn School of Medicine at Mount Sinai, Bronx, USA; 2 Internal Medicine, BronxCare Health System, Bronx, USA; 3 Infectious Disease, BronxCare Health System, Bronx, USA; 4 Pulmonary and Critical Care Medicine, BronxCare Health System, Bronx, USA

**Keywords:** mycobacterium monacense, thoracic spine infection, osteomyelitis, rapid growers of mycobacteria

## Abstract

*Mycobacterium monacense (M. monacense)* is a yellow-pigmented, rapidly growing non-tuberculous mycobacterium (RGM). It is a rare pathogen in humans, and only a very few cases of skin and lung infection related to it have been reported.

In this report, we present the case of a 70-year-old Hispanic male who was brought to the hospital with back pain for 11 months. His physical exam on admission showed point tenderness in the lumbar and thoracic spine. MRI demonstrated severe spinal stenosis, discitis, and adjacent osteomyelitis at the T11-T12 vertebral bodies. Mycobacterium culture with fluorochrome smear from thoracic spine T12 tissue revealed mycobacterium species, but not *Mycobacterium tuberculosis (M. tuberculosis)*. The final culture report led to the identification of *M. monacense*, which was confirmed by DNA sequencing. This case illustrates the rare manifestations of *M. monacense* and highlights the use of molecular biologic techniques to reach a definitive diagnosis in suspected cases.

Infections caused by M. monacense are rarely reported in humans. Even though a few cases have reported *M. monacense* isolated from human samples, the clinical importance of it is not fully understood. A drug susceptibility test for antibiotic therapy is essential for this patient population. The interpretation of these cultures often generates unclear results. However, the aggravation of the disease on imaging and isolation of* M. monacense *alone from the cultured specimens obtained suggested that this pathogen may have caused the infection presented in this case.

## Introduction

*Mycobacterium monacense (M. monacense)* is a yellow-pigmented, rapidly growing non-tuberculous mycobacterium (RGM) [1]. The term *monacense* originated from Monacum, which is the Latin term for Munich, the German city where the first strain was isolated in 2006 by Reischl et al. [1]. The clinically relevant RGM species include *M. chelonae, M. fortuitum, and M. abscessus *subspecies. Bacterial infection due to *M. monacense* is rarely reported in humans. The cases that have been reported so far have revealed that it is usually associated with dermatological and pulmonary infections, especially in immunosuppressed patients [2]. To the best of our knowledge, this is the first case report about thoracic spine osteomyelitis caused by *M. monacense* in the United States. In our patient, the identification of *M. monacense* was confirmed by DNA sequencing.

## Case presentation

A 70-year-old Hispanic man, born in the Dominican Republic, was admitted to the hospital for back pain for 11 months. He stated that the pain had been getting worse for a couple of months before the presentation to the point that he could not ambulate without assistance. The patient had been self-administering over-the-counter pain medication with minimal relief. His other comorbidities included asthma, end-stage renal disease on hemodialysis, hypertension, and insulin-dependent diabetes mellitus. He reported penicillin allergy-causing angioedema. He did not use tobacco, alcohol, illicit drugs, and did not have any recent travel history. In the emergency department, he was ill-appearing and was in mild distress. His vital signs included a body temperature of 36.8 °C (98.3 °F), blood pressure of 146/72 mmHg, a pulse of 77/minute, a respiration rate of 17/minute, and the pulse oximetry showed oxygen saturation of 95% on ambient air. His physical exam on admission showed point tenderness on palpation of the lumbar and thoracic spine but no surrounding erythema, a sensory deficit in the anterior thigh bilaterally, a slow gait, absent bilateral ankle reflexes, negative straight leg raise (SLR) test, and no motor weakness.

The labs revealed leukocyte counts of 6.3 × 10^9^ cells/L, hemoglobin of 8.7 g/dL, platelet counts of 253,000/mm^3^, erythrocyte sedimentation rate (ESR) of 95 mm/hour, C-reactive protein (CRP) of 51.83 mg/L (reference level: <5.0 mg/L), and serum ferritin of 982.0 ng/mL (reference range: 13-150 ng/mL). Serological tests returned positive for surface antibody (anti-HBs), indicating hepatitis B immunization. Blood cultures and mycobacterium culture with fluorochrome smear from respiratory specimens were negative.

**Figure 1:**
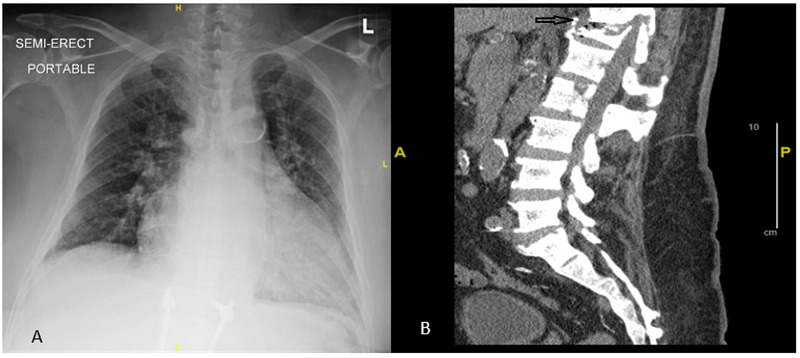
Chest X-ray and lumbar spine CT scan of the patient A: the chest X-ray revealed no active chest disease. B: the lumbar spine CT scan without contrast revealed chronic erosion and sclerosis at the T11-12 with increased loss of height, erosion at the T12, and paraspinal soft tissue thickening, raising concern for acute on chronic osteomyelitis CT: computed tomography

During the past 11 months before the current presentation, the patient had been hospitalized twice for the same reason. Eleven months before the current visit, the patient had presented to the hospital with similar complaints of back pain and fever. His blood culture had isolated methicillin-sensitive* staphylococcus aureus* (MSSA) at that time, which had been treated with appropriate antibiotics. MRI had demonstrated discitis and adjacent osteomyelitis in the T11-T12 vertebral bodies. He had been desensitized to penicillin and had received cefazolin to continue therapy with dialysis. He had been scheduled to receive eight weeks of therapy from the date of the first blood culture without growth. He had had a negative workup for endocarditis with an echocardiogram. Final confirmation of the diagnosis with bone biopsy had not been performed due to the recognition of a clear source of infection, and he had been discharged with the prescription of antibiotics and hemodialysis for eight weeks. He had completed the course of antibiotics with dialysis but had been subsequently lost to follow-up. Six months before the current visit, he had suffered a mechanical fall for which he had visited the emergency department; at that time, lumbosacral spine X-ray had shown degenerative changes, but no acute fracture or spondylolisthesis. On his current visit, he again presented with back pain, which had worsened to the point that he could not ambulate without assistance and required the use of a wheelchair. His chest X-ray showed no infiltrates to suggest active pulmonary pathology (Figure 1A). A lumbar spine CT scan without contrast revealed chronic erosion and sclerosis at the T11-T12 vertebra with increased loss of height and erosion at the T12 vertebra, raising concern for acute on chronic osteomyelitis (Figure 1B).

**Figure 2:**
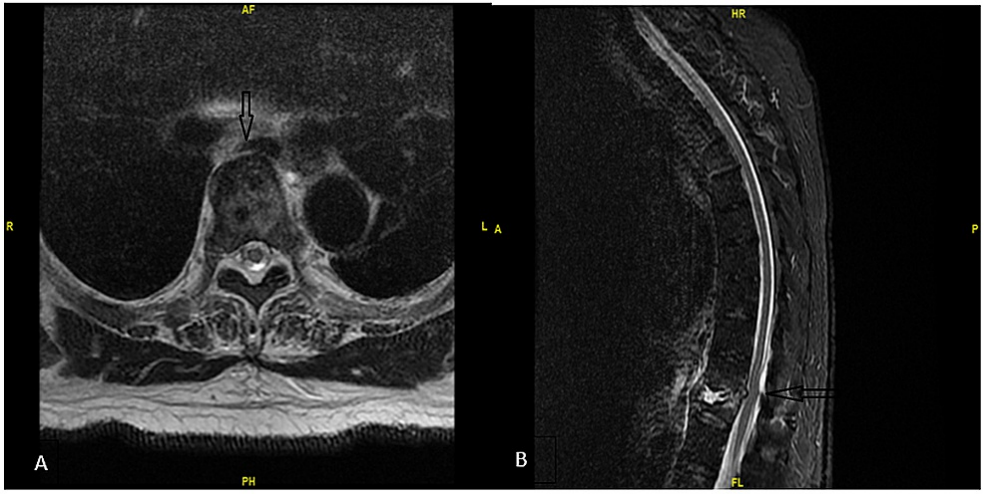
MRI of the thoracic and lumbar spine with and without contrast A: MRI axial view revealed discitis osteomyelitis involving the T11-T12 intervertebral disc space. B: the sagittal view showed there was a collapse of the T12 vertebral body, significant surrounding paravertebral soft tissue, and abnormal enhancing soft tissue was noted surrounding the thecal sac at the level of the T11-T12 vertebra. There may have been an area of significant compression of the thecal sac MRI: magnetic resonance imaging

MRI of the thoracic spine showed osteomyelitis of T11-T12 intervertebral disc space and discitis, which progressed compared to MRI of the thoracic spine one year ago (Figure 2). The infectious disease (ID) department was consulted for acute on chronic discitis and osteomyelitis with a vertebral compression fracture. The patient was suspected of having an MSSA infection based on previous cultures and history. ID recommended sending blood cultures and holding antibiotics until pathological specimens from vertebra were obtained unless the patient experienced clinical deterioration. Neurosurgery was consulted for severe spinal stenosis at T11-T12 vertebra and multilevel disc degeneration. Neurosurgery performed posterior thoracic laminectomy at T11-T12 vertebra, cultures of the disk space and bone biopsy, and posterolateral fusion of T12-L1. Empiric antibiotics with vancomycin and cefepime were started after surgical specimens were obtained. Pathological analysis of the biopsy specimen revealed scant cancellous bone showing sclerosis with remodule change, adjacent fibroconnective tissue hyalinization, and minimal chronic inflammation without any sign of osteomyelitis. AFB staining was not performed. Serum QuantiFERON-TB Gold (QFT) test (Qiagen, Hilden, Germany) returned positive, and mycobacterium culture with fluorochrome smear from thoracic spine T12 tissue revealed mycobacterium species, but not *M. tuberculosis*. The patient was suspected of having *M. tuberculosis* and Pott's disease based on cultures and blood work.

The empiric antibacterial therapy was stopped, and anti-tuberculosis combination therapy with isoniazid, pyrazinamide, rifampin, and ethambutol was initiated while awaiting the DNA probe results for identification of the organism. The final culture report led to the identification of *M. monacense*, which was confirmed by DNA sequencing. The patient continued to undergo therapy with standard anti-tuberculous drugs, pending susceptibility testing. The patient’s culture finding was discussed with the National Jewish Medical and Research Center in Denver regarding the management of *M. monacense*. After a review of the literature, the decision was made to discontinue standard anti-tuberculous therapy, pending results of susceptibility testing. The susceptibility testing report showed that the isolate was sensitive to multiple antibiotics including cefoxitin (8 µg/ml), amikacin (<1 µg/ml), ciprofloxacin (0.25 µg/ml), doxycycline (<0.12 µg/ml), clarithromycin (0.12 µg/ml), imipenem (<2 µg/ml), linezolid (<1 µg/ml), minocycline (<1 µg/ml), tigecycline (0.06 µg/ml), moxifloxacin (<0.25 µg/ml), and trimethoprim/sulfamethoxazole (1/19 µg/ml). The patient showed clinical improvement but did not follow up with the ID clinic for a post-discharge evaluation.

## Discussion

*M. monacense* is a non-tuberculous mycobacterium [1]. It is scotochromogenic, gram-positive, non-motile, acid-fast, produces yellow pigment, and does not form spores in the colonies [1,3]. Infections caused by *M. monacense* are rarely reported in humans, and despite the fact that a few cases have reported *M. monacense* isolated from human samples, its clinical importance is not fully understood. The first strain was isolated in 2006 by Reischl et al. from a bronchial lavage sample in Germany [1]. We conducted a comprehensive literature search with the keywords "Mycobacterium monacense, thoracic spine infection, osteomyelitis, rapid growers of mycobacteria" on PubMed and Google Scholar, which returned a total of nine cases of *M. monacense* associated with human infections [1,2,4-7], with none of them showing osteomyelitis of the thoracic spine. The incidence and prevalence of *M. monacense* are unknown as only a limited number of cases have been reported so far. An epidemiological study from the European Union mentioned only two reported cases, which were from Italy during the period 2001-2010 [8]. Dermatological and soft tissue infections due to *M. monacense* have been reported. *M. monacense* osteomyelitis is a rare condition, but its diagnosis can be made after obtaining bone samples via surgical biopsy; DNA sequencing via polymerase chain reaction (PCR) is mandatory for confirming the diagnosis.

The clinical and laboratory standards and institute guidelines are helpful for treating *M. monacense *infection*. M. monacense* is susceptible to clarithromycin, ciprofloxacin, doxycycline, and amikacin *in vitro* testing [3]. The drug susceptibility test for antibiotic therapy is essential for this patient population. In our case, the patient was initially treated empirically with first-line anti-tuberculosis drugs. However, after the confirmation of the diagnosis, his anti-tuberculosis drugs were stopped pending susceptibility results. The isolate was reported to be susceptible to cefoxitin, amikacin, ciprofloxacin, doxycycline, clarithromycin, imipenem, minocycline, linezolid, tigecycline, moxifloxacin, and trimethoprim/sulfamethoxazole. The interpretation of these cultures is often unclear, and contamination is certainly plausible; However, the evidence of worsening of the condition on imaging and the isolation of *M. monacense* alone from the cultured specimens obtained indicated that it may have been the causal factor behind the infection in our case. After several literature reviews, we have concluded that no other similar case of spinal osteomyelitis caused by *M. monacense, *which was identified and confirmed by DNA sequencing,​​​* *has been reported in the United States. This case illustrates the rare manifestations of *M. monacense *and highlights the use of molecular biologic techniques to make a definitive diagnosis in suspected cases. Unfortunately, the patient did not follow up with the ID clinic for a post-discharge evaluation.

## Conclusions

Infections related to *M. monacense *are rarely reported in humans. Although a few cases have reported *M. monacense* isolated from human samples. its clinical significance is not yet fully understood. The drug susceptibility test for antibiotic therapy is mandatory for this patient population. Even though the interpretation of these cultures can often be unclear, in our case, the worsening of disease on imaging and the fact that only *M. monacense *was isolated from the clinical specimens obtained led us to implicate *M. monacense *as the responsible pathogen.

## References

[1] Reischl U, Melzl H, Kroppenstedt RM (2006). Mycobacterium monacense sp. nov. Int J Syst Evol Microbiol.

[2] Romero JJ, Herrera P, Cartelle M, Barba P, Tello S, Zurita J (2016). Panniculitis caused by Mycobacterium monacense mimicking erythema induratum: a case in Ecuador. New Microbes New Infect.

[3] Tortoli E (2014). Microbiological features and clinical relevance of new species of the genus Mycobacterium. Clin Microbiol Rev.

[4] Hogardt M, Schreff AM, Naumann L, Reischl U, Sing A (2008). Mycobacterium monacense in a patient with a pulmonary tumor. Jpn J Infect Dis.

[5] Taieb A, Ikeguchi R, Yu VL, Rihs JD, Sharma M, Wolfe J, Wollstein R (2008). Mycobacterium monacense: a mycobacterial pathogen that causes infection of the hand. J Hand Surg Am.

[6] Therese KL, Gayathri R, Thiruppathi K, Madhavan HN (2011). First report on isolation of Mycobacterium monacense from sputum specimen in India. Lung India.

[7] Shojaei H, Hashemi A, Heidarieh P, Hosseini N, Daei Naser A (2012). Chronic pulmonary disease due to Mycobacterium monacense infection: the first case from Iran. Ann Lab Med.

[8] van der Werf MJ, Ködmön C, Katalinić-Janković V (2014). Inventory study of non-tuberculous mycobacteria in the European Union. BMC Infect Dis.

